# Microencapsulated Functional Additives in Commercial Diets: Effects on Growth, Health, and Intestinal Pro-Inflammatory Gene Expression in Juvenile Rainbow Trout (*Oncorhynchus mykiss*)

**DOI:** 10.3390/ani16101515

**Published:** 2026-05-15

**Authors:** Elena Antonia Belfiore, Federico Conti, Matteo Zarantoniello, Eleonora Spinozzi, Maria Vittoria Tignani, Riccardo Petrelli, Simone Ceccobelli, Giuliana Parisi, Ike Olivotto

**Affiliations:** 1 Department of Life and Environmental Sciences, Università Politecnica delle Marche, 60131 Ancona, Italy; e.a.belfiore@pm.univpm.it (E.A.B.); federico.conti@staff.univpm.it (F.C.); 2 Department of Agricultural, Food and Environmental Sciences, Università Politecnica delle Marche, 60131 Ancona, Italy; s.ceccobelli@staff.univpm.it; 3 Chemistry Interdisciplinary Project (ChIP) Research Center, School of Pharmacy, University of Camerino, 62032 Camerino, Italy; eleonora.spinozzi@unicam.it (E.S.); riccardo.petrelli@unicam.it (R.P.); 4 Department of Agriculture, Food, Environment and Forestry, University of Florence, 50144 Firenze, Italy; mariavittoria.tignani@unifi.it (M.V.T.); giuliana.parisi@unifi.it (G.P.)

**Keywords:** fish welfare, functional feed additives, microencapsulation, fillet quality, antioxidant status, growth performance

## Abstract

Functional feed additives can improve growth, health, and fillet quality in farmed fish, but their stability during feed processing is limited. This study evaluated the effects of dietary microencapsulated rosemary essential oil (REO), astaxanthin (AX), and butyric acid (BA) on growth, oxidative response, and fillet quality in juvenile rainbow trout (7.9 ± 0.3 g; 90 days). Growth parameters, intestinal and liver health, oxidative status, and fillet pH and color, together with proximate composition and fatty acid profile, were measured. All supplemented groups exhibited higher growth performance compared to controls. Histology and stress/inflammatory markers indicated no adverse effects on tissue health. Regarding fillet quality, REO maintained protein content, AX enhanced lipid content and color stability, and BA promoted a leaner fillet with higher protein deposition. Fillet pH remained more stable in REO and BA groups after frozen storage, suggesting improved product stability. Fatty acid profiles were moderately altered, with REO increasing C18:1n7 and AX enhancing polyunsaturated fatty acids, whereas BA had minimal effects. Plasma antioxidant enzyme activities were lower in supplemented fish, indicating improved redox balance. Overall, microencapsulation preserved the bioactive properties of REO, AX, and BA, supporting their practical use as targeted functional feed additives in rainbow trout aquaculture.

## Simple Summary

Improving fish growth performance and quality of the product is a key goal for modern sustainable aquaculture. This study investigated the effects of adding microencapsulated rosemary essential oil, astaxanthin, and butyric acid to the diet of rainbow trout. These additives are natural compounds known to support growth, gut health, and antioxidant defense. Fish receiving supplemented diets grew faster and showed better body composition, with leaner or more protein-rich fillets depending on the additive. The treatments also improved fillet color. Overall, the results showed that these microencapsulated additives are safe, well-tolerated, and effective in enhancing both fish performance and the nutritional and commercial value of the fillet. Such strategies could contribute to more sustainable, efficient, and consumer-friendly aquaculture practices.

## 1. Introduction

In 2022, global aquaculture production reached a record 130.9 million tonnes, including 94.4 million tonnes of farmed aquatic animals [1]. According to FAO projections, aquaculture production is expected to continue increasing, reaching approximately 104 million tonnes of aquatic animals in 2025, with further moderate growth anticipated in 2026, mainly driven by the expansion of carp, salmon, catfish, shrimp, and other commercially farmed species. For the first time, aquaculture production exceeded that of capture fisheries (91 million tons), evidencing a significant shift in the global supply of aquatic food resources [1]. This milestone has further solidified the position of aquaculture as a fundamental component of global food security. Nevertheless, this rapid expansion has brought significant challenges for this sector (especially considering intensive farming), including increased environmental impacts, rising economic pressures, and the need for more effective and sustainable feed management strategies [2,3,4,5,6]. Particularly, aquafeeds represent a major cost driver in aquaculture operations [2,5,7]. The sector’s reliance on marine-derived raw materials, increasingly limited due to overfishing, has prompted a shift toward alternative protein sources, including meals derived from plant, insects, animal by-products, and microbial biomass [8,9,10]. Among these, plant-based ingredients are widely adopted for their cost-effectiveness and availability [8,11,12,13,14]. However, especially in carnivorous species like salmonids, their high dietary inclusion may impair fish growth, health, and feed intake due to the presence of anti-nutritional factors and low palatability, ultimately affecting water quality through increased organic waste [9,15,16,17].

In addition, intensive aquaculture systems often expose fish to chronic stress due to high stocking densities, suboptimal water quality, and inadequate handling practices [3,18,19,20]. These stressors can affect immune function, which in turn increases disease susceptibility, impairs growth performance, reduces feed efficiency, and elevates mortality rates [21,22,23]. As a result, optimizing both nutritional strategies and rearing practices is essential to safeguard fish health and support the long-term sustainability of aquaculture systems [4,22].

In this context, the health and welfare of farmed fish have emerged as key priorities, with increasing emphasis on the development of tailored diets to meet species-specific needs. Dietary enrichment with targeted nutrients and feed additives has proven effective in enhancing physiological responses, alleviating the effects of stress, and improving overall growth performance and resilience [14,19,24,25,26,27]. A wide range of feed additives are currently employed in aquaculture to preserve nutritional quality, enhance feed processing, stimulate growth, increase feed intake, and supply essential nutrients [28,29]. In particular, feed additives with immunostimulant, antioxidant, and antimicrobial properties, typically incorporated at low inclusion levels, have proven their effectiveness in supporting fish’s overall health status [27,29,30,31]. These additives comprise a broad spectrum of bioactive compounds, including postbiotics (e.g., butyric acid), antioxidants (e.g., astaxanthin), and phytogenic additives derived from essential oils (EOs) of aromatic herbs [27,29,30,31,32,33]. These compounds have been tested in salmonid aquaculture and have been demonstrated to act through multiple mechanisms [31,34,35,36]. Particularly, postbiotics like butyric acid derivatives have been introduced into rainbow trout (*Oncorhynchus mykiss*) diets to strengthen intestinal barrier function and improve nutrient utilization [17,30,37,38,39]. Phytogenic additives, including essential oils from herbs like rosemary and oregano, have also gained attention for their antimicrobial, antioxidant, and anti-inflammatory properties in rainbow trout culture, contributing to improved welfare, growth performance, and oxidative status [31,35,40]. Finally, among feed additives with antioxidant features, astaxanthin has gained attention as a multifunctional compound beyond its role in flesh pigmentation in salmonids. In fact, astaxanthin effectively mitigates oxidative stress by protecting tissues from reactive oxygen species (ROS) and reducing lipid peroxidation, thereby preserving cellular integrity [41,42,43,44,45]. Furthermore, astaxanthin modulates the expression of immune-related genes, enhancing resistance to infections and supporting overall health status [44,46,47,48,49,50].

Despite their beneficial properties, the incorporation of feed additives into aquafeeds often increases the need for proper feed management by the producers and the farmers [6]. In fact, maintaining the stability of these molecules throughout processing, distribution, and storage remains a major challenge, since exposure to heat, moisture, and oxygen can reduce their bioavailability and efficacy in aquatic production systems [14,45,51,52]. Although the beneficial effects of astaxanthin, rosemary essential oil, and butyric acid have been individually described in salmonids, limited information is currently available regarding the efficacy of their microencapsulated forms under practical aquafeed conditions. In particular, there is still a lack of knowledge on whether microencapsulation can effectively preserve the biological activity of these compounds during feed processing and administration, while simultaneously improving fish growth performance, oxidative balance, and fillet quality without compromising fish health. Furthermore, comparative studies evaluating these additives under the same experimental conditions are still scarce.

In this context, preserving the integrity and optimizing the functionality and bio-efficacy of these sensitive molecules is of major importance. The present study aimed to evaluate a natural-based microencapsulation process as an effective strategy for delivering sensitive feed additives in aquafeeds [20,24,26,53]. Microencapsulation is widely recognized in aquaculture nutrition as a tool to protect labile bioactive compounds from degradation (e.g., oxidation and leaching) and to enable controlled release in the gastrointestinal tract, thereby enhancing their biological efficacy [26,53]. Particularly, according to previous studies [24,54,55], three bioactive substances were individually microencapsulated within an organic wall matrix: (i) natural astaxanthin (AX) extracted from *Haematococcus pluvialis* microalgae; (ii) rosemary essential oil (REO) obtained from *Rosmarinus officinalis* L. leaves; and (iii) butyric acid (BA). Following microencapsulation, these compounds were incorporated as feed additives into a commercial diet formulated for rainbow trout juveniles and administered over a 90-day feeding trial. The authors hypothesized that microencapsulation would preserve the bioactive properties of these compounds during feed preparation and storage, thereby enhancing their in vivo efficacy. Specifically, it was expected that dietary supplementation with microencapsulated additives would improve growth performance, modulate oxidative status, and positively affect fillet quality traits without inducing adverse effects on fish health or tissue integrity. Rainbow trout was selected as the model species due to its importance in global aquaculture, accounting for ~25.5% of the European market value and 69.9% of total farmed fish production in Europe [56,57,58].

## 2. Materials and Methods

### 2.1. Ethics

The biological samples of rainbow trout (*O. mykiss*) obtained from the aquaculture facility “Ittica Tranquilli” and used in the present study do not require any ministerial authorization. The samples were obtained exclusively from farmed specimens bred and reared within a licensed aquaculture facility. Therefore, the collection neither involved wild populations nor activities subjected to national or ministerial permits under current regulations.

Accordingly, the sampling procedures comply with all applicable laws and regulations governing aquaculture and the use of farmed aquatic organisms for research and related purposes. Ethical exemption was granted by the Università Politecnica delle Marche (8 January 2026) Ethics Committee. At the end of the trial, a lethal dose (1 g/L) of MS-222 (tricaine methanesulfonate, Merck KGaA, Darmstadt, Germany) was used to minimize animal suffering.

### 2.2. Bioactives

Rosemary EO (REO) was obtained from dried leaves of *R. officinalis* (1 kg; batch no. C-130324-18, harvested on November 2024; origin: Morocco, Minardi & Figli S.r.l., Bagnacavallo, Ravenna, Italy) that were subjected to six-hour hydro-distillation in 10 L of distilled water using a Clevenger-type glass apparatus for the REO collection. The extraction yield, calculated on a dry-weight basis (% *w*/*w*), was 2.2%. After collection, the REO was stored in amber glass vials at 4 °C prior to analyses and microencapsulation. REO was chemically characterized by employing an Agilent 8890 gas chromatograph coupled with a single quadrupole mass spectrometer (5977B MSD, Agilent Technologies, Santa Clara, CA, USA) and equipped with an autosampler (PAL RTC120, CTC Analytics AG, Zwingen, Switzerland). The REO was diluted 1:100 in *n*-hexane and injected in split mode (1:200). The analytical conditions, as well as the identification of the compounds, were performed as previously reported [59].

The REO was also characterized in terms of refractive index and density. Refractive index measurements were carried out at 25 °C employing an Abbe Refractometer (NAR-1T LIQUID, Atago Co., Ltd., Minato-ku, Tokyo, Japan), featuring the refractive index scale and the BRIX scale. Density measurements were performed at 25 °C using a digital densimeter with an oscillating U-tube (DMA-5000M, Anton-Paar, Graz, Austria). The refractive index and density resulted in 1.467 and 0.907 g/mL, respectively.

Natural astaxanthin (AX; AstaReal^®^ L10, AstaReal AB, Nacka, Sweden) and butyric acid (BA; Merck KGaA, Darmstadt, Germany) were commercially sourced and used without further purification. Together with REO, these compounds were subsequently subjected to microencapsulation prior to inclusion in the experimental diets.

### 2.3. Microcapsules’ Composition

**Figure 1:**
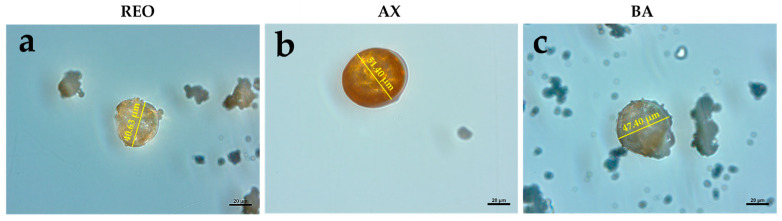
Details of microcapsules observed using a Zeiss optical microscope at 40× magnification: (**a**) REO microcapsules, (**b**) AX microcapsules, and (**c**) BA microcapsules. Scale bar (**a**–**c**): 20 μm. Abbreviations: REO, rosemary essential oil (EO); AX, astaxanthin; BA, butyric acid.

The microcapsules used in this study are identical in composition and were produced using the same microencapsulation process as previously described by Şener et al. [24]. The average size of the microcapsules was 47.51 ± 7.23 µm, exhibiting a smooth and uniform surface (Figure 1). As previously demonstrated by Zarantoniello et al. [55] and Cattaneo et al. [60], this technology ensures strong adhesion of dry microcapsules to any aquafeed particle through well-defined physical and chemical interactions. Moreover, due to their tailored chemical properties, the microcapsules are designed to release their contents approximately 90 s after contact with water, providing sufficient time for ingestion by fish and thereby ensuring effective delivery.

### 2.4. Experimental Diets

A commercial diet for rainbow trout juveniles (NUTRIT 3, 4Fish S.r.l., Via Mercurio 15, 05100 Terni, Italy; https://4fish.net/it/mangime; accessed on 7 January 2025), with pellet size of 3 mm according to the manufacturer’s recommendations, was used as control (CTRL; composition reported in Table 1). The specific composition of the aquafeed used in the present study reported by the company includes marine-derived proteins and lipids: fish meal (high-quality Superprime or LT meal from Atlantic herring, mackerel, blue whiting, and Baltic sprat) and fish oil; plant protein sources: corn gluten meal, dehulled faba bean meal, and alfalfa nutrient concentrate; mineral and functional supplements: monocalcium phosphate, calcified marine algae (*Lithonutrium*), and hydrolyzed yeast; nutritional additives: vitamin and mineral premix (including Vitamin A, Vitamin D3, and Vitamin E). No further details (such as specific amounts) are provided because of industrial property restrictions.” Importantly, all experimental diets were formulated starting from the same commercial basal diet used as CTRL. Therefore, regardless of the background antioxidant content naturally present in the feed or vitamin premix, all treatment groups were exposed to the same basal nutritional and antioxidant matrix. Consequently, the observed differences among treatments can be reasonably attributed to the supplementation with the tested microencapsulated additives rather than to variations in the original diet composition.

Three experimental diets were produced by incorporating microencapsulated REO, AX, or BA in different CTRL diet batches as follows: (i) REO diet: 2.5 g/kg of feed of microencapsulated REO, corresponding to 0.41 g/kg of active REO. This concentration fell within the range previously reported for plant EOs in commercial fish diets (0.1–0.6 g/kg) [61,62]; (ii) AX diet: 4.6 g/kg of feed of microencapsulated AX, corresponding to 0.122 g/kg of pure AX, according to concentrations recommended in previous studies [44]; (iii) BA diet: 12.0 g/kg of microencapsulated BA, corresponding to 0.6 g/kg of butyric acid, in accordance with Magnoni et al. [63]. An additional group receiving the basal diet supplemented with empty microcapsules (CTRL+) was included to account for any potential effects of the encapsulation matrix itself. Since no significant differences were observed compared to the CTRL group in the present study, data obtained from the CTRL+ group are not presented here to maintain focus on the experimental groups of primary interest. In fact, previous investigations similarly demonstrated that empty microcapsules did not have a measurable effect on fish welfare, growth performance, and overall physiological responses [64]. Nonetheless, these data are available upon request.

For each diet, the microcapsules were thoroughly mixed with the CTRL feed pellets in vacuum-sealed glass jars to ensure their uniform distribution. The effective presence and the uniform distribution of all the microcapsules in the experimental diets were verified weekly by analyzing subsamples from each feed (in triplicate for each diet) using a stereomicroscope (Leica Microsystems GmBH, Wetzlar, Germany) equipped with a camera (Invenio 10SCIII, DeltaPix; Smørum, Denmark). In all cases, microscopic observations confirmed that microcapsules remained stably adhered to the feed pellets over time, with no evident detachment or aggregation.

To confirm the quantity of the microencapsulated feed additives, three subsamples of each experimental diet were analyzed at the beginning and at the end of the feeding trial to assess the stability of REO, AX, and BA. Particularly, to verify the presence of REO, three subsamples of the REO diet were analyzed, according to Diab and Thompson [65], and the average REO concentration was 0.38 ± 0.03 g/kg at both sampling times. AX concentration was measured following the methodology described by Du et al. [66]. The average AX concentration was 0.10 ± 0.02 g/kg at both sampling times. Finally, the concentration of butyric acid in the BA diet was assessed according to Kim et al. [67]. The average BA content was 0.58 ± 0.03 g/kg at both sampling times.

### 2.5. Experimental Design

The feeding trial was conducted at the aquaculture facility, Ittica Tranquilli S.r.l. (Corone di Preci, 06047 Preci, PG, Italy). A total of 360 rainbow trout juveniles (initial body weight: 7.9 ± 0.3 g) were randomly assigned to 12 flow-through fiberglass tanks (500 L; 3 tanks per experimental group), according to the different dietary treatments: (i) CTRL group, fish fed the control diet; (ii) REO group, fish fed the REO diet; (iii) AX group, fish fed the AX diet; (iv) BA group, fish fed the BA diet.

Each experimental group consisted of 90 fish (30 fish per tank) and was maintained in outdoor tanks under natural light conditions (the average daily photoperiod was 14 h light/10 h dark). Water quality parameters, including temperature, pH, ammonia, nitrite, and nitrate, were daily monitored throughout the experimental period and remained within optimal ranges for rainbow trout culture over the whole trial (temperature: 13.0 ± 0.5 °C; pH 7.2 ± 0.1; ammonia: <0.05 mg/L; nitrite: <0.05 mg/L; nitrate: <20 mg/L).

Fish were hand-fed the experimental diets at 3% of body weight for 90 days. Feed rations were adjusted every two weeks according to periodic intermediate measurements of biomass increase. Daily visual inspections were performed to monitor fish health and to remove and record any deceased individuals.

At the end of the trial, all fish were euthanized using a lethal dose of anesthetic solution (MS-222, Merck KGaA, Darmstadt, Germany; 1 g/L) and individually weighed. Blood samples (collected from the caudal vein using a heparinized syringe), fillets, distal intestine, and liver were obtained and properly preserved for the following analyses.

### 2.6. Survival Rate, Somatic Growth, and Feed Utilization Indexes

At the end of the 90-day feeding trial, all fish from each tank (90 fish per experimental group) were weighed using an analytical balance (OHAUS Explorer, OHAUS Corp., Greifensee, Switzerland), with an accuracy of 0.1 mg. All administered feed was completely consumed, with no residual feed observed. For each tank, survival rate (SR), weight gain (WG), specific growth rate (SGR), feed conversion ratio (FCR), and daily feed intake (DFI) were calculated as follows:

SR (%) = (final number of fish/initial number of fish) × 100;(1)

WG (%) = [(FBW − IBW)/IBW] × 100;(2)

SGR (% day^−1^) = [(ln FBW − ln IBW)/days of trial] × 100; (3)

FCR = total feed intake (g)/weight gain (g);(4)

DFI (% body weight day^−1^) = [daily feed intake (g)/mean body weight (g)] × 100; (5)

where IBW is the initial body weight and FBW is the final body weight.

### 2.7. Physical Parameters, Chemical Analyses, and Oxidative Status in Fillets

Both right and left fillets from 5 fish per tank (15 per experimental group) were collected. Physical parameters (pH and color) were measured on the left fillet muscle, both immediately after sampling (T0) and after 12 weeks of frozen storage (T1, −20 °C), using a pH meter (Oakton^®^ Acorn™ pH 6, Sigma-Aldrich, St. Louis, MO, USA) and a colorimeter (Minolta CR-200 with illuminant D65, Konica Minolta, Osaka, Japan). More specifically, color, measured in three positions of fillet (cranial, medial, and caudal), was expressed according to the Commission Internationale de l’Éclairage (CIE, 1978) system (L*a*b*), where L* represents lightness, a* is the redness index, and b* is the yellowness index. Successively, both right and left fillets of each fish were lyophilized, ground, and stored at −20 °C.

Considering chemical analyses, fillet samples deriving from the same tank were pooled together (5 fish per pool), obtaining 3 samples per experimental group. Samples were then analyzed following the AOAC procedures [68] for dry matter, crude protein, lipid, and ash content determination. Moisture was obtained by calculating the weight loss after sample lyophilization. Crude protein and lipid contents were determined using the Kjeldahl method (after samples’ acid digestion and using nitrogen-to-protein conversion factor of 6.25; AOAC 981.10) and the Soxhlet ether method (AOAC 991.36), respectively. Finally, ash content was determined through incineration in a muffle furnace by combustion at 550 °C for three hours (AOAC 920.153). For fatty acid profile determination, both right and left fillets from 5 fish per tank (15 fish per experimental group) were collected, freeze-dried, ground, and stored at −20 °C. Total lipid content was extracted according to Folch’s method [69], and the total lipids were then gravimetrically quantified. Fatty acids were determined in the lipid extract after trans-esterification to methyl esters (FAME) using a base-catalyzed trans-esterification [70]. The fatty acid profile was determined by gas chromatography using a Varian GC 430 gas chromatograph (Varian Inc., Palo Alto, CA, USA) equipped with a flame ionization detector and a capillary column (Supelco Omegawax™ 320 m, Supelco^®^, Bellefonte, PA, USA). The resulting chromatograms were recorded with the Galaxie Chromatography Data System 1.9.302.952 (Varian Inc., Palo Alto, CA, USA). Fatty acids were identified by comparing their FAME retention times to those of the Supelco 37 component FAME mix standard (Supelco, Bellefonte, PA, USA) and then quantified using calibration curves, with tricosanoic acid (C23:0) (Supelco, Bellefonte, PA, USA) as an internal standard. The gas chromatography conditions were recovered from Secci et al. [71]. Chromatograms were recorded with the Galaxie Chromatography Data System 1.9.302.952 (Varian Inc., Palo Alto, CA, USA).

Additionally, primary oxidation products were measured in the lipid extract as conjugated dienes (CD), and secondary oxidation products were measured as thiobarbituric acid reactive substances (TBARS) according to the spectrophotometric methods [72,73]. The results were expressed as mmol hydroperoxides (mmol Hp/100 g fillet) and mg of malondialdehyde equivalents (mg MDA-eq./100 g fillet) for CD and TBARS, respectively.

### 2.8. Histological Analysis

Liver and distal intestine samples were collected from 5 fish per tank (15 fish per experimental group) and immediately fixed by immersion in Bouin’s solution (Merck KGaA, Darmstadt, Germany) at 4 °C for 24 h. After fixation, tissues were dehydrated through a graded ethanol series (80%, 95%, and 100%), cleared in xylene (Bio-Optica, Milan, Italy), and embedded in paraffin (Bio-Optica, Milan, Italy). Serial transverse sections, 5 µm thick, were obtained using a microtome (Leica RM2125 RTS, Leica Microsystems GmbH, Nussloch, Germany), and three sections per sample, collected at 200 µm intervals, were selected for analysis. Sections were stained with Mayer’s hematoxylin and eosin Y (HE; Merck KGaA, Darmstadt, Germany) to assess general tissue morphology. Morphometric and histopathological analyses were performed according to Zarantoniello et al. [74], on three transversal sections per fish (15 fish per experimental group). Briefly, in the distal intestine, the mucosal folds’ height and width, as well as the submucosa width, were measured, and the occurrence frequencies of the phenomena reported in Table 2 were assessed. Considering liver, hepatocyte morphology, and degree of fat accumulation in the hepatic parenchyma were assessed. Particularly, the fat fraction was quantified in areas free of blood vessels and bile ducts using ImageJ software (ver. 1.54d; setting a homogeneous threshold value) and expressed as the percentage of total hepatic parenchyma occupied by fat.

### 2.9. Plasma Antioxidant Parameters Analysis

Blood samples were collected from 5 fish per tank (15 fish per experimental group) *via* caudal venipuncture using sodium-heparinized syringes. A new syringe for each fish was used. Blood samples collected from each fish were transferred into tubes containing anticoagulant (heparin) and centrifuged at 5000× *g* for 5 min (MiniSpin G, Sigma-Aldrich, St. Louis, MO, USA) for plasma separation. Plasma was then carefully collected with a pipette, transferred to new tubes, and immediately stored at −80 °C for subsequent analyses.

Superoxide dismutase (SOD) activity (15 plasma samples per group) was determined using the xanthine oxidase/cytochrome c method, as described by Crapo et al. [75]. This assay is based on the spectrophotometric measurement of cytochrome c reduction by the superoxide anion generated through the xanthine oxidase/hypoxanthine reaction. Absorbance was recorded at 550 nm, and measurements were performed using a 10 mm light path cuvette and a nanophotometer(NanoPhotometer^®^ P330, Implen, GmbH, Munich, Germany). Enzymatic activity was expressed as U/mg of protein, where one unit of SOD activity is defined as the amount of sample required to produce 50% inhibition under the assay conditions. The reaction mixture contained 46.5 mM potassium phosphate buffer (pH 7.8), 0.1 mM EDTA, 0.05 mM xanthine, 0.02 mM cytochrome c, and 2.5 µU of xanthine oxidase (XO).

Catalase (CAT) activity was measured following the method described by Aebi [76]. Plasma samples (from 5 fish per tank, 15 per experimental group) were mixed with 30 mM H_2_O_2_ solution (ε = −0.0436 mM^−1^ cm^−1^) and 50 mM phosphate buffer (pH 7.8). The decrease in absorbance was recorded at 240 nm, every 10 s for 3 min, using a 10 mm light-path cuvette and a nanophotometer (NanoPhotometer^®^ P330, Implen, GmbH, Munich, Germany). CAT activity was expressed as U/mg of protein, where one unit of CAT is defined as the amount of enzyme required to catalyze the dismutation of 1 µmol of H_2_O_2_ per minute.

Glutathione peroxidase (GPx) activity (15 plasma samples per experimental group) was measured following the 96-well plate method described in Sattar et al. [77]. Briefly, the reaction medium contained 100 μL of reduced glutathione (500 μM) prepared in phosphate buffer (100 mM; pH = 7.0), and 10 μL of a supernatant. The reaction was started by adding 40 μL of hydrogen peroxide (1.5 mM) and incubating for 10 min at 37 °C. After that, the reaction was stopped with 65 μL of 2% (*w*/*v*) sodium hydrogen phosphate (Na_2_HPO_4_) solution and 35 μL of Elman’s reagent, and the absorbance was measured at 412 nm using a microplate reader (Synergy HTX, Biotek Instruments Inc., Winooski, VT, USA). GPx activity was expressed as U/mg of protein, where one unit of GPx activity was defined as the amount of enzyme required to oxidize 1.0 micromole of reduced glutathione (GSH) to glutathione disulfide (GSSG) per minute under standard conditions of pH 7.0 and temperature of 37 °C [78].

The protein content in plasma samples was determined according to the Bradford method [79] using bovine serum albumin as a standard.

### 2.10. Gene Expression Analysis

Both distal intestine and liver samples were collected from 3 fish per tank (9 fish per experimental group), immediately frozen, and stored at −80 °C until analysis. Total RNA was extracted according to Zarantoniello et al. [55], using RNAzol™ reagent (Merck KGaA, Darmstadt, Germany). Then, to prevent genomic DNA contamination, total RNA samples were treated with DNase (10 IU, 37 °C for 10 min; Thermo Fisher Scientific, Waltham, MA, USA). RNA concentration was determined using a spectrophotometer (NanoPhotometer P-Class, Implen GmBH, Munich, Germany), while RNA integrity was assessed by electrophoresis of 1 μg total RNA stained with GelRed™ on a 1% agarose gel. Purified RNA samples were stored at −80 °C until further use. Complementary DNA was synthesized from 1 μg of total RNA using the iScript™ cDNA Synthesis Kit (Bio-Rad, Hercules, CA, USA). Quantitative Real-Time PCR (qPCR) analyses were conducted using an iQ5 iCycler thermal cycler (Bio-Rad, Hercules, CA, USA), following the reaction setup and cycling conditions reported by Conti et al. [64]. Two no-template controls were included in each run to confirm the absence of contamination, as indicated by the lack of amplification signals. Melting curve analysis revealed a single peak for each amplicon, confirming reaction specificity. qPCR products were sequenced to verify homology.

In liver samples, the expression of genes involved in both stress (glucocorticoid receptor, *nr3c1*; heat shock protein 70, *hsp70*) and oxidative stress (catalase, *cat*; superoxide dismutase, *sod*; and glutathione peroxidase, *gpx1a*) responses was analyzed. In the intestine samples, the expression of genes involved in the intestinal pro-inflammatory response (interleukin 1β, *il1b*, and tumor necrosis factor alpha, *tnf-alpha*) was analyzed. Two reference genes, elongation factor 1-alpha (*ef1α*) and 18S ribosomal protein (*18s*), were employed as internal controls for normalization in both tissues. The primer sequences utilized in the analyses are listed in Table 3. Quantification of mRNA expression was performed using the iQ5 optical system software (version 2.0; Bio-Rad, Hercules, CA, USA) along with the GeneEx Macro iQ5 Conversion and GeneEx Macro iQ5 files.

### 2.11. Statistical Analysis

For survival rate, somatic growth, and feed utilization indexes, tanks were considered as experimental units, whereas individual fish were treated as experimental units for all other analyses. Data normality and homogeneity of variance were evaluated using the Shapiro–Wilk and Levene’s tests, respectively. A one-way analysis of variance (ANOVA) was then performed, followed by Tukey’s post hoc test to compare the mean of each treated group (REO, AX, and BA) with the mean of the CTRL group. Moreover, for fillet physical parameters (pH and color), a paired t-test was used to compare T0 and T1 measurements on the same fillets within each experimental group. Statistical significance was set at *p* < 0.05. All results are reported as mean ± standard deviation (SD). Statistical analyses were carried out using GraphPad Prism 8 software (ver. 8.0.2; San Diego, CA, USA).

## 3. Results

### 3.1. Bioactives: Rosemary Essential Oil (REO) Chemical Characterization

The chemical composition of REO (Table 4) was mainly dominated by oxygenated monoterpenes (58.83%), monoterpene hydrocarbons (19.74%), and monoterpene ketones (19.04%). Minor constituents were sesquiterpene hydrocarbons (0.49%), monoterpene esters (0.32%), and oxygenated sesquiterpenes (0.30%). Among the main constituents, eucalyptol and camphor were the predominant compounds, accounting for 47.66 and 19.04% of the total composition, respectively. Minor compounds were α-pinene (10.42%), borneol (5.00%), and α-terpineol (4.60%).

### 3.2. Survival Rate, Somatic Growth, and Feed Utilization Indexes

Survival rate, somatic growth, and feed utilization index values are reported in Table 5. Survival rate, initial body weight (IBW), feed conversion ratio (FCR), and daily feed intake (DFI) did not differ significantly between the CTRL and each experimental group. In contrast, all treated groups exhibited significantly higher final body weight (FBW), weight gain (WG), and specific growth rate (SGR) than the CTRL group (*p* < 0.05).

### 3.3. Fillet Quality Traits

#### 3.3.1. Fillet Color and pH

Results regarding fillet color parameters (L*, a*, and b*) of rainbow trout fed different experimental diets are reported in Table 6. At T0, L* and a* values did not differ significantly between CTRL and any treated group. Similarly, for b* values, no significant differences were observed between CTRL and REO or BA groups, whereas the AX group exhibited a significantly higher value compared to the CTRL group (*p* < 0.05).

After 12 weeks of frozen storage (T1), none of the treated group showed significant differences in L* values compared to the CTRL group. In contrast, each treated group showed a significantly higher a* value than CTRL (*p* < 0.0001). In addition, yellowness (b*) was significantly lower in both REO and BA groups compared to CTRL *(p* < 0.0001), while the AX group did not show significant differences compared to CTRL.

Comparing L*, a*, and b* values obtained at T0 and T1 within the same dietary group, significant changes over 12 weeks were noted (Table 6; differences indicated by superscript letters). Specifically, all the experimental groups were characterized by a significant increase in L* values (CTRL, *p* < 0.0001; REO, *p* < 0.0001; AX, *p* < 0.0003; BA, *p* < 0.0015) and a parallel significant decrease in a* values (CTRL, *p* < 0.0001; REO, *p* < 0.0001; AX, *p* < 0.0001; BA, *p* < 0.0001) at T1 compared to T0. Finally, in terms of the b* parameter, only CTRL (*p* < 0.0001) and BA (*p* < 0.0001) groups showed a significantly higher value at T1 compared to T0, while no significant differences were detected between the two sampling times considering REO and AX groups.

Table 7 reports the pH values of the fillets. At T0, all the treated groups showed a significantly higher pH value compared with the CTRL group (REO, *p* < 0.0009; AX, *p* < 0.0012; BA, *p* < 0.0005). In contrast, at T1, only the REO (*p* < 0.0001) and BA (*p* = 0.0028) groups were characterized by significantly higher pH values compared to the CTRL, which did not show significant differences with the AX group. Finally, comparing pH values obtained at T0 and T1 within the same dietary group, a significant decrease over a 12-week period was evidenced in all the experimental groups (CTRL, *p* < 0.0001; REO, *p* < 0.0051; AX, *p* < 0.0001; BA, *p* < 0.0001).

#### 3.3.2. Chemical Analysis

Table 8 reports the proximate composition of fillets from rainbow trout juveniles fed the control and the experimental diets. Considering dry matter and moisture content, no significant differences were observed between the CTRL and AX or BA groups, whereas the REO had a significantly higher dry matter content and, consequently, a significantly lower moisture content compared to the CTRL group. Crude protein content was significantly higher in the REO and BA groups compared to the CTRL group. In contrast, crude lipid content significantly increased in the AX group and significantly decreased in the BA group compared to the CTRL, which did not show significant differences with the REO group. Finally, no significant differences in ash content were observed between CTRL and the experimental groups.

Table 9 reports the fatty acid profile of fillet from rainbow trout fed the control and the experimental diets. Among saturated fatty acids (SFA), palmitic acid (C16:0) was the most abundant, followed by stearic acid (C18:0). Both these fatty acids did not differ between each experimental group and CTRL, while myristic (C14:0) was significantly lower in the BA group compared to CTRL.

Regarding monounsaturated fatty acids (MUFA), oleic acid (C18:1n9) was the most abundant. No significant differences were detected between the treated groups and CTRL for oleic (C18:1n9), palmitoleic (C16:1n7), and gadoleic (C20:1n9) acids. Vaccenic acid (C18:1n7) exhibited significantly higher values in the REO and AX groups compared to the CTRL.

For polyunsaturated fatty acids (PUFAs), linoleic acid (C18:2n6) was the most abundant, showing a significantly higher value in the AX group compared to CTRL, whilst no significant differences between CTRL and REO or BA groups were detected. The other predominantly represented PUFAs were the α-linolenic (C18:3n3), eicosapentaenoic (C20:5n3), and docosahexaenoic (C22:6n3) fatty acids, which did not differ significantly between any treated group and the CTRL group. The total amount of minor PUFAs was significantly higher in the AX group than in CTRL. Finally, no significant differences were observed between any treated group and CTRL for total SFA, MUFA, PUFA, n3-PUFA, n6-PUFA, or the n3/n6 ratio.

#### 3.3.3. Fillets’ Oxidative Status

Considering conjugated dienes (CD), only the REO group exhibited significantly higher values compared to CTRL, whereas no differences were detected between CTRL and AX or BA groups (Table 10). No significant differences were observed between any treated group and the CTRL in terms of TBARS levels.

### 3.4. Histological Analysis

**Figure 2:**
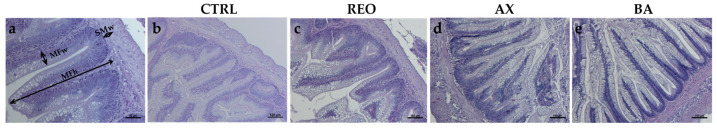
(**a**) Measurement criteria for submucosa width (SMw), mucosal folds height (MFh), and mucosal folds width (MFw). (**b**–**e**) Example of histomorphology of the distal intestine of rainbow trout juveniles fed experimental diets. Scale bars: (**a**) 50 μm; (**b**–**e**) 100 μm.

**Figure 3:**
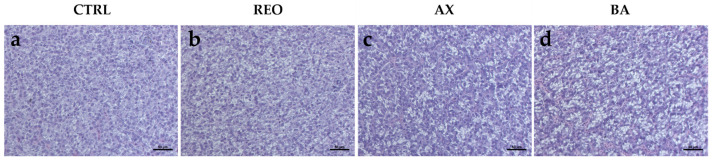
(**a**–**d**) Example of liver histomorphology from rainbow trout fed the experimental diets. Scale bars: (**a**–**d**) 50 μm.

Histological examination did not reveal any pathological changes or inflammatory events in the intestine or liver of fish from any of the experimental groups, indicating a normal tissue architecture (Figure 2 and Figure 3). Accordingly, none of the treated group differed from CTRL in either morphometric measurements (submucosal width and mucosal fold height and width) or histopathological indices, measured in the distal intestine (Table 11 and Figure 2).

Considering the liver (Figure 3), fish from all experimental groups were characterized by a physiological hepatic parenchyma, with no evidence of pathological alterations. In addition, the analysis of the hepatic fat fraction showed no significant differences between any treated group and the CTRL (20.37 ± 1.56, 19.02 ± 3.05, 24.97 ± 3.68, and 16.70 ± 3.24% for CTRL, REO, AX, and BA, respectively).

### 3.5. Plasma Antioxidant Parameters

**Figure 4:**
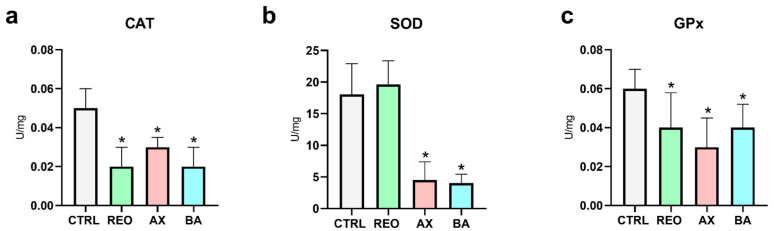
(**a**) Catalase activity (CAT), (**b**) superoxide dismutase activity (SOD), and (**c**) glutathione peroxidase (GPx) activity analyzed in plasma of rainbow trout juveniles fed the experimental diets. Data are presented as mean ± SD (*n* = 15). Asterisks indicate a statistically significant difference between each treated group and the CTRL group (*p* < 0.05).

Catalase (CAT) activity (Figure 4a) was significantly lower in the REO, AX, and BA groups compared to CTRL (*p* < 0.05). Superoxide dismutase (SOD) activity (Figure 4b) was significantly lower in the AX and BA groups than in CTRL (*p* < 0.05), whilst no significant differences were observed between REO and CTRL. Finally, glutathione peroxidase (GPx) activity (Figure 4c) was significantly lower in each treated group compared to CTRL (*p* < 0.05).

### 3.6. Real-Time PCR Results

**Figure 5:**
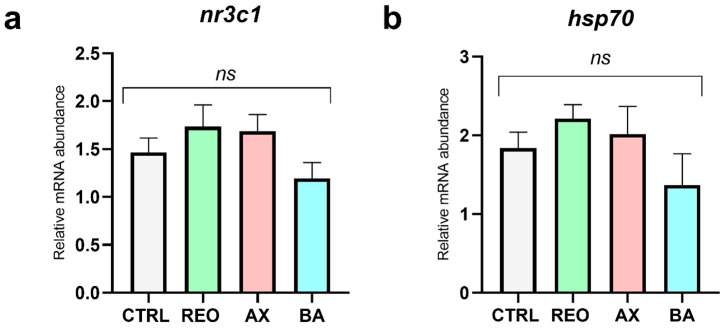
Relative mRNA abundance of stress-related genes (**a**) *nr3c1* and (**b**) *hsp70* in the liver of rainbow trout juveniles fed the experimental diets. Data are presented as mean ± SD (*n* = 9). Abbreviations: *ns*, no significant differences.

The relative mRNA abundance of the stress-related genes *nr3c1* and *hsp70* in the liver of juvenile rainbow trout did not differ significantly between the experimental groups and the CTRL (Figure 5a,b).

**Figure 6:**
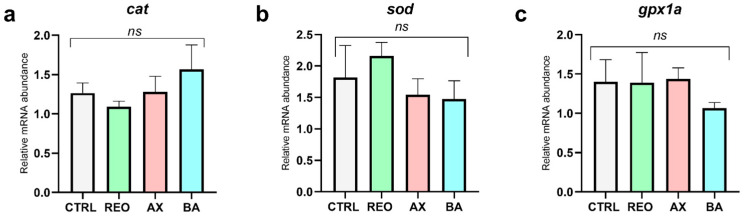
Relative mRNA abundance of antioxidant-related genes, (**a**) *cat* (catalase), (**b**) *sod* (superoxide dismutase), and (**c**) *gpx1a* (glutathione peroxidase), analyzed in the liver of rainbow trout juveniles fed the experimental diets. Data are presented as mean ± SD. Abbreviations: *ns*, no significant differences.

Similarly, considering the expression of genes involved in the antioxidant response (Figure 6), no significant differences were observed between each experimental group and the CTRL group (*p* > 0.05) for all the genes considered.

**Figure 7:**
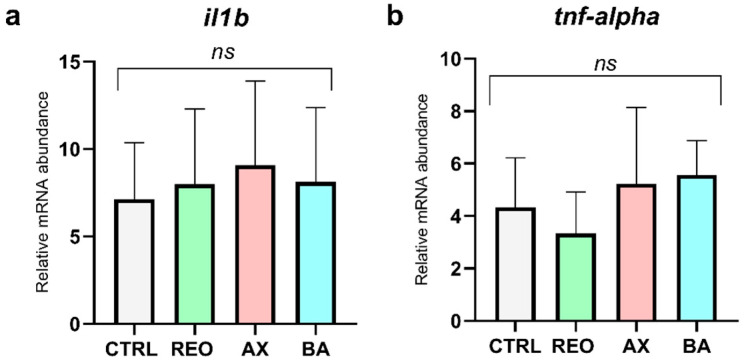
Relative mRNA abundance of (**a**) *il1b* and (**b**) *tnf-alpha* in the distal intestine of rainbow trout juveniles fed the experimental diets. Data are presented as mean ± SD. Abbreviations: *ns*, no significant differences.

Finally, as regards intestinal pro-inflammatory-related markers investigated in the distal intestine (Figure 7), no significant differences were observed between the experimental groups and the CTRL group in the expression of either *il1b* and *tnf-alpha*.

## 4. Discussion

The present study evaluated the efficacy of a natural-based microencapsulation process for delivering sensitive feed additives in aquafeeds, specifically assessing the effects of REO, AX, and BA on growth performance, physiological status, oxidative balance, and fillet chemical properties of rainbow trout juveniles. The chemical composition of REO found in this study resulted in a linear relationship to those reported for other Moroccan, Italian, Iranian, Tunisian, and Turkish REOs [83,84,85,86]. Indeed, eucalyptol was reported as the predominant compound, accounting for more than 40% of the total EO composition [87]. Conversely, the REO composition obtained from our analysis is in contrast with those of Greek, French, and Spanish REOs, for which eucalyptol, camphor, and α-pinene were reported in similar amounts (20–30%) [87]. The variability in the chemical composition of REO is due to several factors, such as geographical origin, climatic conditions, or harvesting [88].

Overall, the results demonstrated that these functional additives, regardless of delivery method, enhanced fish performance and product quality (pH and color) without compromising animal health, confirming their effectiveness as previously reported and highlighting the efficiency of microencapsulation for aquafeed supplementation [35,38,89]. From a physiological perspective, the inclusion of microencapsulated supplements was well tolerated. Histological analyses revealed no alterations in key organs involved in nutrient metabolism, such as the intestine and liver, indicating that none of the dietary treatments impaired organ integrity. This observation was further supported by the absence of differences in the expression of stress-related (*nr3c1* and *hsp70*) and intestinal pro-inflammatory markers (*il1b* and *tnf-alpha*), suggesting that the treatments did not induce physiological stress or local inflammatory responses. These findings confirm the safety of the tested inclusion levels and are consistent with previous studies reporting good tolerability of phytogenics, carotenoids, and postbiotics in salmonids [35,90,91,92].

A major outcome of the study was the significant improvement in growth performance observed in all supplemented groups. Fish from treated groups exhibited higher final body weight, weight gain, and specific growth rate than the CTRL. While FCR and DFI did not differ significantly, FCR tended to be lower and DFI slightly higher in supplemented groups, suggesting that cumulative effects over the 90-day feeding period may have contributed to enhanced growth performance. The mechanisms underlying these improvements were not directly investigated in the present study. However, previous studies have reported that BA may support intestinal health and nutrient absorption [93,94], REO may contribute to digestive functionality [35,95], and AX has been associated with improved metabolic and mitochondrial efficiency [96,97]. In addition, other authors have suggested that these compounds may influence digestive processes, gut integrity, and nutrient utilization efficiency [38,90,91,94]. Therefore, the enhanced growth observed in the present study could be associated with some of these previously described effects, although further investigations including digestive enzyme activity, digestibility measurements, and detailed intestinal morphometry are needed to confirm the underlying mechanisms.

Given the antioxidant properties of some additives, fish oxidative status was also assessed. AX is a potent scavenger of reactive oxygen species (ROS), protecting cellular structures from oxidative damage [96,98,99], while REO, rich in monoterpenes such as α-pinene and eucalyptol, provides free radical-scavenging and lipid peroxidation-protective effects [35,92,95,100,101]. BA contributes indirectly by enhancing gut integrity and mucosal barrier function [90,91], potentially reducing ROS production and supporting digestive efficiency [102]. In this study, hepatic gene expression of antioxidant enzymes (*cat*, *sod*, *gpx1a*) did not differ among treatments, suggesting that fish were not exposed to sufficient oxidative stress levels to trigger transcriptional responses. In contrast, plasma antioxidant enzyme activities (CAT, SOD, GPx) were generally lower in the supplemented groups than in the CTRL. This apparent discrepancy between gene expression and enzyme activity likely reflects post-transcriptional regulation and tissue-specific responses [103,104,105]. The lower plasma enzyme activities may indicate a reduced need for endogenous enzymatic detoxification, as dietary antioxidants directly mitigated ROS accumulation [92,98,106]. Under lower oxidative stress, antioxidant enzymes may be less activated because exogenous antioxidants neutralize reactive species before endogenous defense pathways are triggered. This may explain why supplemented fish maintained oxidative balance despite reduced circulating enzyme activity. Similar patterns have been observed in teleosts fed AX-supplemented diets, where decreased SOD, CAT, and GPx activities were associated with effective ROS scavenging rather than diminished redox balance [106,107,108]. Comparable mechanisms may also apply to REO and BA, although specific evidence in fish remains limited [36,52,109,110].

*Post-mortem* fillet quality is another critical consideration, as long-term storage at low temperatures can reduce product quality by promoting lipid oxidation, protein denaturation, and moisture loss. Dietary supplementation with REO, AX, and BA distinctly influenced fillet composition during frozen storage. REO preserved both crude protein and dry matter content, suggesting potential protective effects against oxidative protein degradation during storage [101]. However, fillets from the REO group also showed higher conjugated diene (CD) levels than the CTRL, indicating increased formation of primary lipid oxidation products. This finding firstly suggests that the protective effect of REO on oxidative stability was not consistent across all lipid oxidation markers. However, TBARS values, which reflect secondary lipid oxidation products, did not differ among groups. Therefore, the increase in CD may represent an early or transient stage of lipid oxidation that did not progress to the formation of secondary oxidation compounds during storage or overall fillet deterioration [111,112,113]. Several mechanisms may explain this response. First, REO is rich in antioxidant compounds such as eucalyptol and α-pinene, which may exert pro-oxidant effects under specific conditions or concentrations [114]. Second, the release kinetics of REO bioactive compounds may have influenced the interaction between antioxidants and unsaturated lipids during frozen storage [115,116]. Overall, although REO supplementation appeared to preserve some fillet quality traits, the data indicate a complex oxidative response that warrants further investigation, particularly regarding dose optimization, release dynamics of encapsulated EOs, and the evolution of oxidation products during storage [111,115,116].

AX, as a lipophilic antioxidant, was associated with higher lipid content, reflecting its protective effect against lipid oxidation and contribution to flesh preservation [117,118]. BA increased crude protein while reducing lipid content, indicating a shift toward lean tissue deposition, although post-mortem lipid losses from oxidation or catabolism cannot be excluded. These findings align with Yarahmadi et al. [119] who reported enhanced protein content and fillet quality in rainbow trout fed sodium butyrate. Overall, the additives influenced nutrient partitioning in fillets through both metabolic modulation *infra vitam* and protective effects during storage. However, no significant differences were observed in cumulative SFAs, MUFAs, PUFAs, or n-3/n-6 ratio, likely due to the use of a high-quality, uniform commercial diet across all groups. Fillet color and pH, key indicators of post-harvest changes, were also affected by supplementation. At harvest (T0), only AX increased the yellowness index (b*), reflecting carotenoid deposition during the grow-out period [89,120,121,122]. After frozen storage (T1), all supplemented groups maintained a higher redness index (a*) compared to the CTRL, particularly AX, supporting the role of dietary carotenoids in pigment preservation and oxidative protection [89,121,123]. Lower yellowness in REO and BA groups at T1 may reflect differences in pigment stability during storage, requiring further investigation [96,98].

*Post-mortem* pH decline, driven by the dissociation of carbonic acid and hydrogen ion release [124], was observed in all groups, although treated groups maintained higher initial pH at T0 and throughout storage [118,125]. These results fell within a more desirable range (closer to 7.0) for fish fillets, reflecting greater stability of final products [118]. This highlighted the potential of functional feed additives to modulate post-harvest fillet quality, a critical factor for sensory acceptance and marketability.

Overall, microencapsulated REO, AX, and BA enhanced growth performance, modulated systemic redox balance, and did not impair fillet quality traits in rainbow trout, while preserving organ integrity and overall fish health. The bioactive properties of these compounds were retained post-encapsulation, confirming the effectiveness of this delivery system and its potential as a practical, flexible tool for targeted feed supplementation at the farm level [126,127].

## 5. Conclusions

This study demonstrated that microencapsulated rosemary essential oil, astaxanthin, and butyric acid can be safely incorporated into juvenile rainbow trout diets. These additives enhanced growth performance and modulated systemic redox balance without compromising organ integrity or overall fish health. Microencapsulation preserved the bioactive properties of the compounds, enabling effective in vivo delivery. Growth benefits may be associated with improved nutrient utilization and digestive efficiency, while reduced plasma antioxidant enzyme activity in the absence of transcriptional changes suggested a possible direct ROS-scavenging effect. The tested additives also exerted distinct effects on fillet characteristics. REO contributed to the maintenance of protein content and post-mortem pH stability, AX was associated with improved color stability and higher lipid preservation during storage, whereas BA promoted a leaner fillet profile with higher protein deposition. However, the effects on fillet quality parameters were treatment-specific rather than uniformly positive across all evaluated indicators. Overall, microencapsulation represents a practical strategy for targeted supplementation, allowing a single-base diet to be customized with bioactive compounds for controlled release while reducing feed management complexity. A limitation of the present study is that the additives were evaluated individually and under controlled experimental conditions over a relatively short production period. Therefore, future studies should optimize inclusion levels, evaluate long-term effects under commercial farming conditions, and explore potential synergistic interactions among additives.

## Figures and Tables

**Table 1:** Proximate composition (%) and energy content (MJ/kg) of the commercial diet (NUTRIT 3, 4Fish S.r.l.) for rainbow trout juveniles used as the control diet in the present study.

CTRL Diet	
Crude protein (%)	45.00
Crude fat (%)	22.00
Crude fiber (%)	1.50
Ash (%)	9.00
Total phosphorus (%)	1.10
Gross energy (MJ/kg)	22.20
Digestible energy (MJ/kg)	20.20

**Table 2:** Score assignment criteria for episodes of mucosal folds fusion, presence of supranuclear vacuoles in enterocytes, and lymphocyte infiltration in the submucosa in the distal intestine of rainbow trout fed the experimental diets.

Index	Scores	Frequency
Episodes of mucosal folds fusion (observations per section)	+	0–5
	++	5–15
	+++	>15
Supranuclear vacuoles	−	Absent
	+	Scattered
	++	Diffused
	+++	Highly abundant
Lymphocyte infiltration	+	Scarce
	++	Moderated
	+++	Diffused

**Table 3:** Primer sequences used in this study.

Genes	Forward Sequence (5′–3′)	Reverse Sequence (5′–3′)	AT (°C)	NCBI ID
*nr3c1*	AGAAGCCTGTTTTTGGCCTGTA	AGATGCGCTCGACATCCCTGAT	58.3	Z54210
*hsp70*	CCCTGGGCATCGAAACC	CCCTCGTAGACCTGGATCATG	58.3	AY423555
*sod*	TGAAGGCTGTTTGCGTGCTGAC	CCGTTGGTGTTGTCTCCGAAGG	58.3	NM_001160614.1
*cat*	CCGTCCTTCGTCCACTCTCAGA	CTCGGCATCCTCAGGCTTCAAG	58.3	XM_021564302.2
*gpx1a*	TCATCATGTGGAGCCCTGTCTG	TCTGCCTCAATGTCACTGGTCA	58.3	HE687021.1
*il1b*	ACATTGCCAACCTCATCATCG	TTGAGCAGGTCCTTGTCCTTG	58.3	NM_001124347.2
*tnf-alpha*	GGCGAGCATACCACTCCTCTGA	AGCTGGAACACTGCACCAAGGT	59.4	NM_001124357.1
*18s* (hk)	CGGAGGTTCGAAGACGATCA	TCGCTAGTTGGCATCGTTTAT	59.4	XR_005038417.1
*ef1α* (hk)	CAACGATATCCGTCGTGGCA	ACAGCGAAACGACCAAGAGG	59.4	AF498320

NCBI identification numbers (IDs) and their respective annealing temperatures (AT). Abbreviations: hk, housekeeping genes.

**Table 4:** Chemical composition of rosemary essential oil (REO).

	Compound ^a^	RI ^b^	RI Lit. ^c^	Area % ± SD ^d^	ID ^e^
1	*α*-pinene	930	932	10.42 ± 0.16	Std,RI,MS
2	camphene	944	946	2.91 ± 0.05	RI,MS
3	*β*-pinene	973	974	0.99 ± 0.01	Std,RI,MS
4	myrcene	989	988	0.66 ± 0.01	Std,RI,MS
5	*α*-phellandrene	1002	1002	0.06 ± 0.00	Std,RI,MS
6	*α*-terpinene	1014	1014	0.22 ± 0.00	Std,RI,MS
7	*p*-cymene	1022	1020	2.62 ± 0.03	Std,RI,MS
8	limonene	1026	1024	1.71 ± 0.08	Std,RI,MS
9	eucalyptol	1028	1026	47.66 ± 0.11	Std,RI,MS
10	*γ*-terpinene	1056	1054	0.07 ± 0.00	Std,RI,MS
11	terpinolene	1086	1086	0.07 ± 0.00	Std,RI,MS
12	linalool	1098	1095	0.80 ± 0.03	Std,RI,MS
13	camphor	1140	1141	19.04 ± 0.07	RI,MS
14	borneol	1162	1165	5.00 ± 0.01	RI,MS
15	terpinen-4-ol	1174	1174	0.78 ± 0.00	RI,MS
16	*α*-terpineol	1187	1186	4.60 ± 0.01	RI,MS
17	bornyl acetate	1283	1287	0.32 ± 0.00	RI,MS
18	(*E*)-caryophyllene	1416	1417	0.49 ± 0.01	Std,RI,MS
					
	Total identified			99.84 ± 0.01	
	Monoterpene hydrocarbons			19.74 ± 0.15	
	Sesquiterpene hydrocarbons			0.49 ± 0.01	
	Oxygenated monoterpenes			58.83 ± 0.15	
	Monoterpene ketones			19.04 ± 0.07	
	Monoterpene esters			0.32 ± 0.00	
	Oxygenated sesquiterpenes			0.30 ± 0.02	

^a^ Components are ordered according to their elution from the HP-5MS column. ^b^ Linear retention index calculated according to the Van den Dool and Kratz formula. ^c^ Retention index taken from Adams. ^d^ Relative percentage values are the mean of two independent analyses (SD, standard deviation). ^e^ Identification methods: Std, comparison with available analytical standard; RI, coherence of the calculated RI with those stored in the ADAMS (2007) [80] and NIST 17 (2017) [81] libraries; MS, mass spectrum matching with respect to ADAMS, FFNSC (2012) [82], and NIST 17 MS libraries.

**Table 5:** Survival rate, somatic growth, and feed utilization indexes of rainbow trout juveniles fed the experimental diets.

	CTRL	REO	AX	BA	*p*-Value
Survival (%)	100	100	100	100	n.d.
IBW (g)	7.8 ± 0.3	7.6 ± 0.2	8.13 ± 0.3	8.0 ± 0.3	0.1553
FBW (g)	53.7 ± 2.5	67.1 ± 2.0 *	66.9 ± 3.0 *	60.3 ± 3.2 *	0.0009
WG (%)	588.5 ± 20.0	782.9 ± 31.0 *	722.9 ± 27.2 *	653.7 ± 24.3 *	<0.0001
FCR	1.01 ± 0.05	0.91 ± 0.06	0.93 ± 0.04	0.92 ± 0.04	0.1163
SGR (% day^−1^)	2.07 ± 0.03	2.34 ± 0.02 *	2.27 ± 0.03 *	2.17 ± 0.03 *	<0.0001
DFI (% BW day^−1^)	1.54 ± 0.08	1.64 ± 0.09	1.62 ± 0.10	1.57 ± 0.08	0.5220

Data are presented as mean ± SD (*n* = 3). IBW, initial body weight; FBW, final body weight; WG, weight gain; FCR, feed conversion ratio; SGR, specific growth rate; DFI, daily feed intake. Within each row, an asterisk indicates a statistically significant difference between each treated group and the CTRL (*p* < 0.05).

**Table 6:** Color parameters (L*, a*, and b*) changes in fillets from rainbow trout juveniles fed the experimental diets, analyzed at T0 (fresh, immediately after slaughtering) and at T1 (after frozen storage at −20 °C for 12 weeks).

	CTRL		REO		AX		BA	
	T0	T1	T0	T1	T0	T1	T0	T1
L*	46.71 ± 1.79 ^a^	49.65 ± 1.21 ^b^	47.62 ± 0.52 ^a^	51.31 ± 1.58 ^b^	47.06 ± 0.56 ^a^	49.40 ± 1.20 ^b^	47.33 ± 0.88 ^a^	49.43 ± 3.08 ^b^
a*	2.16 ± 0.46 ^b^	−1.56 ± 0.44 ^a^	2.61 ± 1.04 ^b^	−0.68 ± 0.14 ^a^*	2.79 ± 1.19 ^b^	0.48 ± 0.23 ^a^*	2.44 ± 0.92 ^b^	−0.26 ± 0.92 ^a^*
b*	2.50 ± 0.49 ^a^	3.81 ± 0.54 ^b^	2.56 ± 0.55 ^a^	2.60 ± 0.53 ^a^*	3.62 ± 1.06 ^a^*	4.13 ± 0.26 ^a^	2.61 ± 0.63 ^a^	3.18 ± 0.38 ^b^*

Values are expressed as mean ± SD (*n* = 15). For each color parameter, asterisk indicates statistically significant differences between each treated group and the CTRL group within the same sampling time (*p* < 0.05); different superscript letters (a, b) indicate statistically significant differences between T0 and T1 within the same dietary group (*p* < 0.05).

**Table 7:** Fillet pH values of rainbow trout juveniles fed the experimental diets, measured at T0 (fresh, immediately after slaughtering) and at T1 (after frozen storage at −20 °C for 12 weeks).

	CTRL		REO		AX		BA	
	T0	T1	T0	T1	T0	T1	T0	T1
pH	6.73 ± 0.43 ^a^	6.21 ± 0.54 ^b^	7.10 ± 0.15 ^a^*	6.75 ± 0.22 ^b^*	7.09 ± 0.22 ^a^*	6.44 ± 0.22 ^b^	7.12 ± 0.15 ^a^*	6.61 ± 0.09 ^b^*

Data are expressed as mean ± SD (*n* = 15). Asterisks indicate statistically significant differences between each treated group and the CTRL group at the same sampling time (*p* < 0.05). Different superscript letters (a, b) indicate statistically significant differences between T0 and T1 within the same dietary group (*p* < 0.05).

**Table 8:** Proximate composition (%) of fillets from rainbow trout juveniles fed the experimental diets.

	CTRL	REO	AX	BA	*p*-Value
Dry matter (%)	25.99 ± 1.27	27.45 ± 0.14 *	27.05 ± 2.08	26.90 ± 0.65	0.0194
Moisture (%)	74.01 ± 1.27	72.55 ± 0.14 *	72.95 ± 2.08	73.10 ± 0.65	0.0194
Crude protein (%)	17.93 ± 0.49	18.60 ± 0.13 *	18.18 ± 0.13	18.89 ± 0.69 *	<0.0001
Crude lipid (%)	6.77 ± 0.42	6.80 ± 0.36	7.08 ± 0.34 *	6.34 ± 0.14 *	0.0026
Ashes (%)	1.67 ± 0.12	1.66 ± 0.12	1.62 ± 0.14	1.70 ± 0.02	0.2699

Values are expressed as mean ± SD (*n* = 3). Within each row, an asterisk indicates a statistically significant difference between each treated group and the CTRL group (*p* < 0.05).

**Table 9:** Fatty acid profile (% of total FAME) and n3/n6 ratio of fillets from rainbow trout juveniles fed the experimental diets.

	CTRL	REO	AX	BA	*p*-Value
C14:0	2.25 ± 0.16	2.24 ± 0.08	2.14 ± 0.15	2.13 ± 0.10 *	0.0153
C16:0	17.1 ± 0.58	17.2 ± 0.37	16.9 ± 0.10	16.8 ± 0.40	0.0335
C18:0	4.20 ± 0.09	4.39 ± 0.18	4.22 ± 0.09	4.30 ± 0.18	0.9998
other SFA	0.986 ± 0.09	0.998 ± 0.04	0.956 ± 0.04	0.934 ± 0.05	0.1865
C16:1n7	3.33 ± 0.15	3.26 ± 0.11	3.22 ± 0.13	3.21 ± 0.19	0.1198
C18:1n9	28.0 ± 0.77	28.6 ± 0.29	28.5 ± 0.63	28.3 ± 0.76	0.1374
C18:1n7	2.34 ± 0.02	2.38 ± 0.02 *	2.37 ± 0.02 *	2.36 ± 0.03	0.0001
C20:1n9	2.07 ± 0.3	1.90 ± 0.1	1.93 ± 0.03	1.93 ± 0.08	0.8119
other MUFA	2.31 ± 0.16	2.20 ± 0.14	2.28 ± 0.04	2.21 ± 0.15	0.0703
C18:2n6	21.4 ± 0.39	21.6 ± 0.43	22.1 ± 0.10 *	21.7 ± 0.49	<0.0001
C18:3n3	2.09 ± 0.05	2.12 ± 0.04	2.17 ± 0.04	2.15 ± 0.07	0.9304
C20:5n3	1.56 ± 0.14	1.43 ± 0.19	1.47 ± 0.13	1.49 ± 0.12	0.3484
C22:6n3	6.93 ± 0.83	6.15 ± 0.37	6.41 ± 0.67	6.84 ± 0.82	0.1186
other PUFA	5.81 ± 0.08	5.52 ± 0.45	5.39 ± 0.19 *	5.62 ± 0.47	0.0114
∑ SFA	24.5 ± 0.50	24.9 ± 0.54	24.2 ± 0.23	24.2 ± 0.60	0.1265
∑ MUFA	37.8 ± 0.91	38.3 ± 0.28	38.3 ± 0.82	38.0 ± 1.10	0.2865
∑ PUFA	37.6 ± 0.61	36.7 ± 0.95	37.4 ± 0.95	37.7 ± 1.43	0.0434
∑ n3-PUFA	12.0 ± 0.81	11.1 ± 1.05	11.5 ± 0.80	12.0 ± 1.04	0.9999
∑ n6-PUFA	25.3 ± 0.34	25.3 ± 0.54	25.6 ± 0.25	25.5 ± 0.54	0.1627
n3/n6	0.47 ± 0.03	0.44 ± 0.09	0.44 ± 0.02	0.46 ± 0.03	0.2435

Data are expressed as mean ± SD (*n* = 15). Within each row, an asterisk indicates a statistically significant difference between each treated group and the CTRL group (*p* < 0.05). Abbreviations: SFA, saturated fatty acids; MUFA, monounsaturated fatty acids; PUFA, polyunsaturated fatty acids.

**Table 10:** Conjugated dienes (CD) and thiobarbituric acid reactive substances (TBARS) were measured in fillets from rainbow trout juveniles fed the experimental diets.

	CTRL	REO	AX	BA	*p*-Value
CD	0.146 ± 0.040	0.174 ± 0.005 *	0.160 ± 0.010	0.154 ± 0.020	0.0053
TBARS	0.0500 ± 0.030	0.0710 ± 0.040	0.0526 ± 0.010	0.0396 ± 0.010	0.3311

Data are expressed as mean ± SD (*n* = 15). Within each row, an asterisk indicates a statistically significant difference between each treated group and the CTRL (*p* < 0.05).

**Table 11:** Morphometric measurements and histopathological indices were conducted on distal intestine samples of rainbow trout juveniles fed the experimental diets.

	CTRL	REO	AX	BA	*p*-Value
SMw (µm)	68.66 ± 21.67	50.33 ± 9.81	68.64 ± 22.57	67.27 ± 7.10	0.3724
MFw (µm)	57.26 ± 4.04	54.50 ± 6.80	53.23 ± 6.21	53.28 ± 4.67	0.7130
MFh (µm)	535.9 ± 77.46	429.50 ± 35.19	524.10 ± 57.75	523.80 ± 71.27	0.1082
Supranuclear vacuoles	+	+	+	+	n.d.
Lymphocyte infiltration	+	+	+	+	n.d.
Mucosal folds fusion	+	+	+	+	n.d.

Data for mucosal fold height (MFh), mucosal fold width (MFw), and submucosal width (SMw) are expressed as mean ± SD (*n* = 15). Semi-quantitative scoring system used for histological evaluation: episodes of mucosal fold fusion were scored as + (0–5 observations per section), ++ (>5–15 observations per section), and +++ (> 15 observations per section). Supranuclear vacuoles were scored as: − (absent), + (scattered), ++ (diffuse), and +++ (highly abundant). Lymphocyte infiltration was scored as: + (scarce), ++ (moderate), and +++ (diffuse).

## Data Availability

The data presented in the current study are available from the corresponding author on a reasonable request.

## Abbreviations

The following abbreviations are used in this manuscript:

a*	Redness (CIELab color parameter)
ANOVA	Analysis of variance
AOAC	Association of Official Analytical Chemists
AX	Astaxanthin
b*	Yellowness (CIELab color parameter)
BA	Butyric acid
BHT	Butylated hydroxytoluene
CAT	Catalase
CD	Conjugated dienes
CIE	Commission Internationale de l’Éclairage
CL	Crude lipid
CP	Crude protein
CTRL	Control
DHA	Docosahexaenoic acid
DFI	Daily feed intake
EDTA	Ethylenediaminetetraacetic acid
EPA	Eicosapentaenoic acid
FAO	Food and Agriculture Organization
FAME	Fatty acid methyl esters
FBW	Final body weight
FCR	Feed conversion ratio
GPx	Glutathione peroxidase
HE	Hematoxylin and eosin
